# Adequacy of Current State Setbacks for Directional High-Volume Hydraulic Fracturing in the Marcellus, Barnett, and Niobrara Shale Plays

**DOI:** 10.1289/ehp.1510547

**Published:** 2016-02-19

**Authors:** Marsha Haley, Michael McCawley, Anne C. Epstein, Bob Arrington, Elizabeth Ferrell Bjerke

**Affiliations:** 1 Department of Radiation Oncology, University of Pittsburgh Cancer Institute, Pittsburgh, Pennsylvania, USA; 2 School of Public Health, West Virginia University, Morgantown, West Virginia, USA; 3 Department of Internal Medicine, Texas Tech University Health Sciences Center School of Medicine, Lubbock, Texas, USA; 4 Parachute, Colorado, USA; 5 Graduate School of Public Health, Department of Health Policy and Management, University of Pittsburgh School of Law, Pittsburgh, Pennsylvania, USA

## Abstract

**Background:**

There is an increasing awareness of the multiple potential pathways leading to human health risks from hydraulic fracturing. Setback distances are a legislative method to mitigate potential risks.

**Objectives:**

We attempted to determine whether legal setback distances between well-pad sites and the public are adequate in three shale plays.

**Methods:**

We reviewed geography, current statutes and regulations, evacuations, thermal modeling, air pollution studies, and vapor cloud modeling within the Marcellus, Barnett, and Niobrara Shale Plays.

**Discussion:**

The evidence suggests that presently utilized setbacks may leave the public vulnerable to explosions, radiant heat, toxic gas clouds, and air pollution from hydraulic fracturing activities.

**Conclusions:**

Our results suggest that setbacks may not be sufficient to reduce potential threats to human health in areas where hydraulic fracturing occurs. It is more likely that a combination of reasonable setbacks with controls for other sources of pollution associated with the process will be required.

**Citation:**

Haley M, McCawley M, Epstein AC, Arrington B, Bjerke EF. 2016. Adequacy of current state setbacks for directional high-volume hydraulic fracturing in the Marcellus, Barnett, and Niobrara Shale Plays. Environ Health Perspect 124:1323–1333; http://dx.doi.org/10.1289/ehp.1510547

## Introduction

Hydraulic fracturing, also referred to as “fracking,” is a relatively recent well-stimulation technique used in some forms of oil and gas development. The method entails injecting pressurized liquids into rock formations of low permeability (e.g., shale) to mobilize oil or gas to the wellbore (Gandossi 2013). Hydraulic fracturing is used with other novel technologies, such as directional drilling, to access previously inaccessible resources such as shale gas, which has become an increasingly large portion of the overall energy supply in the United States (Pless 2012). Directional drilling increased from 6% of new hydraulically fractured wells drilled in the United States in 2000 to 42% of new wells drilled in 2010 (Gallegos and Varela 2015). This number is rising and the trajectory is expected to continue. A decade ago, shale gas production accounted for 2% of total U.S. output. In 2014, that figure was 37%, and an Information Handling Services study projects that natural gas developed through the use of hydraulic fracturing will rise to more than 75% of the domestic supply by 2035 (API 2014).

As a result of the proliferation of hydraulic fracturing, there is an increasing awareness of the multiple potential pathways leading to human health risks from this practice. Air pollution is a significant pathway: From volatile organic compounds (VOCs) found naturally in shale gas released during the drilling process, during blowdowns and venting (Macey et al. 2014), and through leaks at multiple connection points (U.S. EPA 2014); heavy diesel equipment used in the drilling process (Macey et al. 2014); chemical mixtures used to facilitate extraction (Goldstein et al. 2014); and silica sand as proppant (American Public Health Association 2012). Vapor dispersion is another health concern (Center for Chemical Process Safety 2015); in addition, natural gas well sites have experienced blowouts and other types of explosions (Hoffman 2015).

What constitutes a judicious setback distance between natural gas industrial activities and natural or anthropogenic structures is a debatable issue in more densely populated areas (Begos 2014). The literature is currently lacking concerning which particular setbacks are adequate to protect the health and safety of the public. In this paper we examine setback distances in three states located in three major shale plays—the Barnett, the Marcellus, and the Niobrara—and attempt to determine whether these legal setbacks are adequate.

## Methods

We chose three of the largest and most heavily drilled areas of technically recoverable natural gas resources (natural gas plays) in the United States: the Barnett, Marcellus, and Niobrara (U.S. EIA 2011a), and confined our study to gas wells within three states in these regions of interest. Texas, Pennsylvania, and Colorado were selected to allow a comparison between state setback laws. We used the definition of “gas well” as defined by the International Association of Oil and Gas Producers (OGP) as one which has an estimated gas:oil ratio of > 1,000 (OGP 2010). We first reviewed the intended purpose of setbacks and the distances utilized. We then conducted an analysis of federal and state laws in Texas, Pennsylvania, and Colorado. In addition, Texas had municipal ordinances in place that were preempted by state law in 2015, and these were examined as well.

To determine whether current setbacks provide adequate distance in the case of a well blowout, we compiled historical blowout incidents and evacuations within the Texas Barnett Shale, the Pennsylvania Marcellus Shale, and the Colorado Niobrara Shale. Measurable evacuation zones in adjacent states within the target shale plays were included if available. We used the definition of “blowout” from the OGP as “an incident where formation fluid flows out of the well or between formation layers after all the predefined technical well barriers or the activation of the same has failed” (OGP 2010). In our analysis, we included Level 3 blowout events, which are defined as those that present serious and immediate risks to personnel, equipment, and the environment, and warrant the immediate activation of an emergency response plan. Surface blowouts and underground blowouts with insufficient casing fall into this category (Wild Well Control, Inc., and Travelers Indemnity Company 2012). We compiled the data using state agency reports, literature sources, incident reports, and media reports from 1997 to 2015. Wherever possible, we reviewed multiple reports of the same event to determine consistency and veracity. This search revealed 16 relevant sources, which are referenced in the Results section. We recorded the number of homes/families displaced, using these terms interchangeably. Evacuation zones were reported in feet and/or miles (Table 1). We did not use individual evacuees or well workers in our mathematical data, but discussed them where appropriate.

**Table 1:** Analysis parameters, methods, and units of measurement.

Parameter	Methods	Units
Thermal exposure	Modeling	kW/m^2^
Vapor dispersion (hydrogen sulfide)	Measurements and modeling (literature review)	Concentration (ppm) and distance
Vapor dispersion (carbon disulfide)	Measurements (literature review)	Concentration (mg/m^3^) and distance
Air pollution (benzene)	Measurements (literature review)	Concentration (μg/m^3^ and ppb)

Since natural gas is composed primarily of methane hydrocarbon, it is flammable within a certain range in air (Cashdollar et al. 1996). An ignition source at a natural gas well site has the potential to set off an explosion (Nguyen 2010). Hazard assessment studies from liquefied natural gas fires indicate the potential for thermal injury to humans from radiant heat (Raj 2008). At a well site, if the combustion process occurs in the open air, the gas will burn at a constant pressure, allowing the gas to expand during the process (Arrington 2014). To estimate the radiant heat effects on humans from a natural gas well fire, we applied thermal modeling to a typical gas well. Allowing for a constant pressure and changing volume, the adiabatic flame temperature of methane is 1,950°C (3,452°F). We applied the Stefan-Boltzmann Law to a typical gas well producing 5.8 million ft^3^/day with a pipe diameter up to 6 in. An average well is producing 549 times the fuel needed to supply a 1 ft^2^ flux area. This assumes a flame ball of 549 ft^2^, metric conversion of 51 m^2^, with reduction of 1 m^2^ to allow for a standard industry claim of 98% efficiency decline for a flare (Arrington 2014).

In addition to blowouts and radiant heat, potential hazards from hydraulic fracturing include vapor and toxic gas clouds. Shale gas often contains tens or hundreds of parts per million (ppm) of hydrogen sulfide (H_2_S) (Weiland and Hatcher 2012), a flammable gas with known adverse respiratory and nervous systems effects [Agency for Toxic Substances and Disease Registry (ATSDR) 2014]. We included one recent (2014) reference each from Texas, Pennsylvania, and Colorado on H_2_S measured in proximity to natural gas wells. We reviewed a 2005 report that was prepared for the U.S. Department of Transportation (DOT), Office of Pipeline Safety detailing the potential impact radius (PIR), which can be obtained to determine the possible impact on people or property in the case of failure of natural gas infrastructure (U.S. DOT 2005). A series of best-fit equations were used to relate release rate to distance to toxic end points based on information presented in the U.S. Environmental Protection Agency (EPA) Risk Management guidance document (U.S. EPA 2015b), assuming a 10-min peak-release period (U.S. DOT 2005). We also reviewed a 2011 report by the Fort Worth League of Neighborhoods. The League convened a committee of scientific and health professionals to review air testing data in the vicinity of gas drilling activities in the Barnett Shale. Their report included data from private tests by GD Air Testing Inc., Texas Commission on Environmental Quality (TCEQ 2010), and the Barnett Shale Energy Education Council’s industry-funded study conducted by Titan Engineering (Barnett Shale Energy Education Council 2010). Dispersion modeling was performed to predict the way pollutants might travel from their source (Fort Worth League of Neighborhoods 2011). We used the results from these two studies to determine whether current setback distances provide adequate distance from clinically significant sulfide exposure, based on OSHA and NIOSH adult short-term exposure regulatory and recommended limits (U.S. Department of Labor 2015). Hydrogen sulfide levels are reported in ppm and carbon disulfide levels are reported in milligrams per cubic meter (mg/m^3^) (Table 1).

Air pollution sources from shale gas extraction and its related activities include emissions from engines powering the drilling and hydraulic fracturing operations, equipment used to capture and transport the gas on site, venting, blowdowns, and flaring. Air pollutants include particulate matter, carbon monoxide, nitrogen dioxide, methane, and VOCs (Lattanzio 2013). Notable among the list of VOCs are the BTEX (Benzene, Toluene, Ethyl benzene and isomers of Xylene) compounds, which tend to be found ubiquitously at drill sites (Leusch and Bartlow 2010). In an exploratory study, benzene was the most common BTEX to exceed health-based risk levels (Macey et al. 2014). In addition, benzene is well-studied with regard to deleterious effects on humans (CDC 2013). We therefore focused on benzene for our air pollution analysis. Benzene levels are reported in both parts per billion (ppb) and micrograms per cubic meter (μg/m^3^) to allow comparison between studies (Table 1).

We did not include data from predominantly oil sites, pipeline explosions, or compressor stations. Although we used Occupational Safety & Health Administration (OSHA) and National Institute for Occupational Safety and Health (NIOSH) data (U.S. Department of Labor 2015), we did not include studies of occupational safety and exposure. We did not address drinking well, aquifer, and natural water contamination by formation fluids and hydraulic fracturing fluid.

## Results

### Geography and Production

The Barnett Shale, the largest natural gas play in Texas (Airhart 2015), is located in the north-central part of the state, extending over a total area of 5,000 mi^2^. It lies below the surface of 25 counties in Texas, 4 of these being core counties with the highest gas production (Railroad Commission of Texas 2015a). The Barnett shale produces primarily methane, and the producing gas-oil ratio in the core areas of the Barnett shale is above 100,000 standard ft^3^/stock tank barrels (Holme 2013). There are approximately 18,000 to 19,000 producing gas wells in the Barnett Shale (Barnett Shale Energy Education Council 2012); the majority of these are horizontal wells that employ hydraulic fracturing (U.S. EIA 2011b).

The Marcellus Shale covers 95,000 mi^2^ and stretches across eight states: New York (which currently has a hydraulic fracturing ban) (Klopott 2015); Pennsylvania (which has the most drilling in the Marcellus Shale) (Penn State Public Broadcasting 2014); West Virginia, Ohio, Maryland, and smaller portions of Virginia, Tennessee, and Kentucky. The shale play covers an estimated 64% of Pennsylvania (Curtis 2011), approximately 29,500 mi^2^. The Marcellus is a predominantly methane-producing shale play (Holme 2013). By 2012, Marcellus Shale drilling had affected 0.07% of the total land area of the state (Penn State Public Broadcasting 2014). In 2013, Pennsylvania had over 57,000 producing gas wells; the majority of new wells drilled in Pennsylvania are directional (U.S. EIA 2015).

The Niobrara Shale is situated in Northeastern and Northwestern Colorado and also covers portions of adjacent Wyoming, Nebraska, and Kansas. Natural gas is produced primarily from the Piceance Basin and gas and oil from the Denver-Julesburg (D-J) Basin (Higley and Cox 2007); it is one of the top 10 sources of natural gas in the United States (U.S. EIA 2009). There are approximately 15,000 gas wells in the Colorado Niobrara (Colorado Geological Survey 2011). Over 90% of new gas wells in Colorado use hydraulic fracturing (Weiner 2014).

### Policies and Oversight

Natural gas well setbacks are determined at the state and, in some cases, municipality level (the exception to this is when drilling occurs near public work projects, such as dams and critical structures; in these cases federal regulation applies) (Fry 2013). In general, the source for a setback distance is considered to be the well bore, although this is not specifically indicated in all statutes. As discussed below, setback distances vary among the three states we studied (Table 2), and all three have variances which can shorten the distance.

**Table 2:** Legal setback distances by state.

State	Minimum setback distance from buildings without variance
Texas	200 ft
Pennsylvania	500 ft
Colorado	500 ft (1,000 ft high occupancy)

Within the Barnett Shale of Texas, setbacks are designed to protect the health, safety, and welfare of residents; protect the rights of property owners; safeguard environmental quality; and promote efficient gas extraction. The Railroad Commission of Texas (RRC) is responsible for activities associated with oil and gas, including exploration, extraction, production, and transport (Fry 2013). The RRC does not directly determine setback distances; per Texas State Legislature Section 253.005c, a well “may not be drilled in the thickly settled part of the municipality or within 200 feet of a private residence” (Texas State Legislature 2009). In Texas, variances are granted “to prevent waste or to prevent the confiscation of property” (RRC 2015c). The majority of applications for gas drilling in the Dallas/Fort Worth Metroplex area contain a distance setback variance request (Welch 2015). Many municipalities consider the minimum setback to be too close and have established local setback distances. For example, setback rules vary among the 26 municipalities in heavily drilled Denton County, with a range of 300–1,500 ft, mode of 1,000 ft. With variance, the range is 150–1,125 ft, mode of 300 ft (Fry 2013). Recently, the state of Texas passed into law H.B. No. 40, which preempts regulation of oil and gas operations by municipalities (Texas State Legislature 2015); therefore all sites will presumably be required to conform to state law—even those such as the city of Denton, which had previously banned hydraulic fracturing entirely.

In Pennsylvania, setback distances are determined by the state legislature and enforced primarily by the Pennsylvania Department of Environmental Protection (PA DEP 2014b). In February of 2012, the Pennsylvania General Assembly enacted the Omnibus Amendment to the Oil and Gas Act (commonly known as Act 13), intended to strengthen environmental standards for unconventional shale gas extraction (Pennsylvania General Assembly 2012). According to Title 58, Section 3215 of the Pennsylvania Legislature, the current setback distance to buildings is 500 ft, unless the owner of the structure consents to a shorter distance (Pennsylvania General Assembly 2016). PA DEP may grant a variance from these distance restrictions if the well operator submits a plan identifying additional measures. Also, existing active well sites are “grandfathered” in and new wells can be drilled closer than 500 ft from a dwelling at such sites (PA DEP 2014b).

In Colorado, setbacks are determined by the Colorado Oil and Gas Conservation Commission (COGCC). The stated purpose of setbacks is to “protect the safety and welfare of the general public from environmental and nuisance impacts resulting from oil and gas development in Colorado, including spills, odors, noise, dust, and lighting” (COGCC 2013). In 2013, 2 CCR 404-1 Cause No. 1R Docket No. 1211-RM-04 established new rules for statewide setbacks (COGCC 2013). The distance is 500 ft from buildings (such as homes and commercial facilities), 1,000 ft from high-occupancy buildings (schools, day care centers, hospitals, nursing homes, and correctional facilities), 350 ft from outdoor recreational areas (playgrounds and sports fields), and 150 ft from a surface property line. Energy companies are also expected to employ mitigation measures to reduce the impact of their operations upon the public. Variances may be granted for existing wells, if the operator employs mitigation reassures, or if alternate locations are technically or economically impractical (COGCC 2013).

Federal laws provide for clean air (U.S. EPA 2015d); however, with few exceptions, natural gas extraction activities are exempt from these laws (NRDC 2013). Under federal law, gas well operators must comply with Title 40 of the Code of Federal Regulations, which outline emission standards and compliance schedules for the control of VOCs and sulfur dioxide (SO_2_) emissions (U.S. EPA 2012b). The United States Environmental Protection Agency (EPA) requires gas well operators to utilize green completions (capturing of excess gas instead of releasing it into the atmosphere) to reduce air pollution from VOCs (U.S. EPA 2012a). According to Title 40 Subpart 0000 §60.5375, if state rules are more stringent and do not otherwise conflict with federal regulations, state law will prevail (U.S. EPA 2012b). In Texas, air quality is managed by TCEQ (RRC 2015a). In Pennsylvania, the PA DEP has the authority to regulate air quality, and operators are required to utilize detection and repair methods to control volatile organic compounds and associated hazardous air pollutants (PA DEP 2014a). In Colorado, emissions are overseen by the Colorado Department of Public Health and Environment (2013).

Thermal exposure criteria are regulated on a national basis. The National Fire Protection Association (NFPA; see http://www.nfpa.org/about-nfpa) is a global nonprofit organization which sets standards to eliminate death, injury, property and economic loss due to fire, electrical and related hazards. Liquid Natural Gas (LNG) Standard, NFPA 59A, sets limits in terms of maximum heat flux. For human outdoor exposure the limiting heat flux is 5 kW/m^2^ (kilowatt per square meter) (NFPA 2015). The thermal radiation protection requirements in the U.S. Department of Transportation Regulations in 49 CFR, part 193 (U.S. GPO 2015) specify essentially the same requirements as NFPA 59A. The U.S. Department of Housing and Urban Development (HUD) regulations, which are applicable to HUD-assisted residential projects, have a much lower threshold of 1.4 kW/m^2^ (HUD 1982).

Raw natural gas contains hydrogen sulfide (H_2_S), which is classified by the EPA as a hazardous air pollutant (U.S. EPA 2015d). Due to its toxicity, flammability, and corrosive properties, H_2_S is an important component to control at all stages of natural gas handling. H_2_S has destructive effects on natural gas extraction and transportation equipment; there is also a threat to personnel working at natural gas sites (Ratner and Tiemann 2015). The U.S. Department of Labor recommends well-site management based on potential exposure to H_2_S. OSHA set a ceiling limit of 20 ppm for hydrogen sulfide in workplace air, which is a 15-min time-weighted average that cannot be exceeded at any time during the working day. NIOSH recommends a 10-min ceiling level of 10 ppm for workers; 100 ppm is immediately dangerous to life or health of workers (U.S. Department of Labor 2015). A Minimal Risk Level of 0.07 ppm has been recommended by the ATSDR for acute-duration inhalation exposure to hydrogen sulfide, and a Minimal Risk Level of 0.02 ppm has been derived for intermediate-duration inhalation. Death has occurred after acute exposure to high concentrations (≥ 500 ppm) of hydrogen sulfide gas (ATSDR 2014). Carbon disulfide is another sulfide compound with neurotoxicological properties. OSHA 15-min exposure limit is 36 mg/m^3^, and NIOSH 15-min limit is 30 mg/m^3^ (ATSDR 1996).

### Blowouts and Evacuations

Within the Barnett Shale between 1997 and 2006, there were 18 well blowouts—14 blowouts in Wise County and 4 in Denton County (Nguyen 2010). Since 2006, 16 blowouts have been reported by operators (RRC 2015b). A blowout in 2002 forced the evacuation of 30 homes in Haslet, TX (Nguyen 2010). In December 2005, an operator lost control of a Barnett Shale gas well in Palo Pinto County. The ensuing explosion blew a 750-ft-wide crater in the ground, and the fire burned uncontrollably for several days (Heinkel-Wolfe 2013; Nguyen 2010). On 22 April 2006, a blowout in Fort Worth required evacuation of 500 homes in a ½-mi radius. One worker was killed (Korosec 2006; Nguyen 2010). On 19 April 2013, a gas well blowout required evacuation of four homes and diversion of flights from the Denton Enterprise Airport (Heinkel-Wolfe 2013). On 11 April 2015, uncontrolled pressurized flowback required the evacuation of 100 homes and an evacuation zone of ⅛ mi (Arlington Voice 2015). On 7 May, lightning struck a gas well in Denton, resulting in an explosion and fire. No evacuation was ordered, but residents self-evacuated due to overwhelming smoke and fumes (Sakelaris 2015).

In June of 2010, a blowout in the Marcellus Shale of Clearfield County, Pennsylvania, spewed gas and drilling fluid 75 ft into the air, requiring closure of roads and a no-fly zone over the area. No evacuations were needed as there were no homes within 1 mi (Hurdle 2010; Nguyen 2010). On 7 June 2010, an explosion at a Moundsville, West Virginia, Marcellus shale well required burn unit hospitalization for seven people and closure of a highway (Nguyen 2010; Templeton and Hopey 2010). In April of 2011, a well blowout in Bradford County required evacuation of seven families (Casselman 2011). In June of 2012, a blowout in Tioga County required a 1-mi evacuation zone, with contingent plans for a 2-mi zone in case the well could not be brought under control (Detrow 2012). In March of 2013, a blowout in Wyoming County required a 1,500 ft evacuation zone and evacuation of three families (Legere 2013). On 11 February 2014, three gas wells exploded at a gas well site in Dunkard Township, Green County, Pa. The fire burned for 5 days, and well control was not regained until 2 weeks after the explosion. The accident killed one gas well worker and injured another. A ½-mi safety perimeter was established around the pad (RKR Hess 2014). At this rural site, no homes or businesses required evacuation (Santoni 2014). In September 2014, a blowout in Mercer County caused an evacuation of homes within a 1-mi radius of the well pad (CBS Pittsburgh 2014). In October 2014, a well rupture in adjacent Jefferson County, Ohio, Marcellus required evacuation of 400 families (Arenschield 2014).

In April of 2012, the operator lost control of a gas well in the Niobrara Shale of Wyoming, requiring evacuation of 67 residents within a 2.5 mi radius (Gebrekidan and Schneyer 2012).

### Thermal Modeling

Damage from well-pad fires is a function of time and energy flux intensity and, in general, damage increases the longer a fire burns. In addition, the interval between blowout and gas ignition can affect the size of the resulting fireball and the extent of explosive damage. At a well site, if the combustion process occurs in the open air, the gas will burn at a constant pressure, allowing the gas to expand during the process (Arrington 2014). The risks to people and objects outside a vapor cloud fire arise primarily from radiant heat emitted by the fire (Raj 2007).

Applying the Stefan-Boltzmann Law to a typical gas well as described in the Methods section, at 500 ft the thermal exposure would be 2.98 kW/m^2^; at 350 ft the thermal exposure would be 6 kW/m^2^ (Arrington 2014).

### Vapor Dispersion

Measurements of H_2_S in four core counties in the Barnett Shale showed that 8.0% of wells had hydrogen sulfide concentrations > 4.7 ppb (0.0047 ppm) beyond the fence line (Eapi et al. 2014). PA DEP has designated 19 wells as “Special Caution Areas” due to elevated levels of H_2_S encountered during drilling, defined as > 20 ppm (PA DEP 2014b), which is above the 15-min OSHA ceiling limit (U.S. Department of Labor 2015). In a community–based grab sample study, one in five samples in Colorado contained H_2_S that exceeded ATSDR intermediate minimal risk levels (Macey et al. 2014).

PIR calculations presented in the U.S. DOT report resulted in a hydrogen sulfide toxic gas cloud radius of 0.27 mi (1,426 ft) for urban conditions and 0.37 mi (1,954 ft) for rural conditions (U.S. DOT 2005).

In the report by the Fort Worth League of Neighborhoods (2011) described in the Methods section, various sulfur compounds were detected at extremely high levels. The neurotoxin carbon disulfide was found at levels 300 times the norm for ambient urban air. Based on the testing results, dispersion modeling was performed for a drill site near an elementary school. The carbon disulfide plume extended 1 mi from the source; the full extent of plume was in excess of 2 mi. The model predicted up to 1,000 times the short term health benchmark for carbon disulfide, based on OSHA and NIOSH adult short-term exposure regulatory and recommended limits (ATSDR 1996). A second model on carbonyl sulfide was performed based on a site near three elementary schools and one high school. The plume extended in excess of 1 mi, with levels six times the health benchmark for carbonyl sulfide (Fort Worth League of Neighborhoods 2011).

### Air Pollution

Within the Barnett Shale, air quality canister sampling identified 70 individual volatile organic compounds in the vicinity of gas wells and associated transport operation. The most abundant non-methane VOCs, accounting for approximately 90% of emissions, were ethane, propane, butane, and pentanes (Kibble et al. 2013). In 2009, TCEQ used infrared cameras to survey 94 natural gas sites in the Dallas-Fort Worth area in order to identify potential sources of emissions (Whiteley and Doty 2010). Air samples were collected at 73 of the sites; at 21 of those sites, benzene levels exceeded the U.S. EPA level for long-term health effects (ATSDR 2007), and 2 sites required immediate action for benzene levels high enough to pose an immediate threat to health and safety (Ethridge 2010). In 2010, testing by TCEQ confirmed that toluene and carbon disulfide, in addition to other chemicals, were being emitted by gas facilities in the Barnett Shale. Their report concluded that “gas production facilities can, and in some cases do, emit contaminants in amounts that could be deemed unsafe” and that “35 chemicals were detected above appropriate short term comparisons” (TCEQ 2010; Fort Worth League of Neighborhoods 2011).

**Figure 1:**
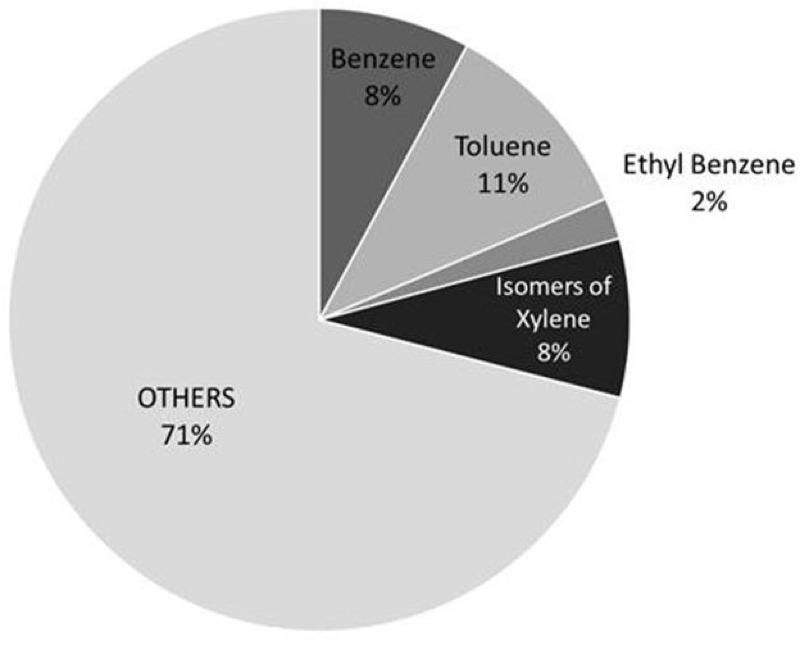
Distribution of chemical species of VOCs around Marcellus Shale drill sites. Michael McCawley. Air Contaminants Associated with Potential Respiratory Effects from Unconventional Resource Development Activities. Seminars in Respiratory and Critical Care Med 2015;36:379–387, Thieme Publishers, www.thieme.com (printed by permission).

In a community-based study in Susquehanna County, Pennsylvania, 25% of grab samples from well pads and associated infrastructure contained benzene levels that exceeded the 1/100,000 U.S. EPA cancer risk level (Macey et al. 2014; U.S. EPA 2015c). McCawley, working for the West Virginia Department of Environmental Protection in May 2013, obtained air samples 625 ft from the well pad center at seven unconventional drilling sites in the West Virginia Marcellus, specifically for the purpose of determining if the 625 ft setback established for West Virginia was adequate to protect public health (McCawley 2013). Five of the sites were locations of active drilling and completion activities, and two sites involved only site preparation work. There were 22 data points provided, 15 of which came from the five active sites, and 7 of which came from the two well-pad preparation sites, all located 625 ft away from the well pad center. Benzene was found at the highest concentration of any of the VOCs, although toluene was the single VOC found most frequently (Figure 1) (McCawley 2015). Benzene levels exceeded the ATSDR minimum risk level for acute exposure-9 ppb (28.7 μg/m^3^) for exposure of 14 days or less—in 5 out of 15 samples, and at 3 out of the 5 active drilling sites. The two highest benzene values, 85 ppb (270 μg/m^3^) and 49 ppb (160 μg/m^3^), were found at a single site during hydraulic fracturing and flowback activities. Well-pad preparation was not associated with elevated benzene levels (McCawley 2013).

In Colorado, daily air samples collected by the National Oceanic and Atmospheric Administration Boulder Atmospheric Observatory revealed that oil and gas activities, including shale gas extraction, were strongly associated with alkane and benzene levels in the atmosphere (Pétron et al. 2012). McKenzie et al. (2012) performed a health risk assessment by analyzing samples collected by the Garfield County Department of Public Health and Antero Resources. In 2008, the Garfield County Department of Public Health collected ambient air well completion samples, including emissions from both uncontrolled flowback and supporting completion equipment such as trucks and generators. Samples were taken 130–500 ft from the well pad. In 2010, Antero Resources Inc. collected ambient air samples 350 and 500 ft from the well pad center during completion activities. No other hydrocarbon sources were in the vicinity of the sampling locations. These samples were compared with 163 samples taken from a fixed monitor in a rural natural gas development area 2,500 ft away from the nearest well pad. The median air level of benzene in the well completion samples was 2.6 μg/m^3^ (0.82 ppb), which is below level of concern, but benzene samples were found to be highly variable: the 95% level of benzene was 20 μg/m^3^ (6.26 ppb), which is right at the 6 ppb Minimal Risk Level for intermediate exposure (ATSDR 2007), and the maximum benzene level was 69 μg/m^3^ (21.6 ppb), which is more than twice the 9 ppb minimal risk level for acute exposure (ATSDR 2007). The benzene levels in the natural gas development area, by contrast, never reached levels of concern for health impacts. Residents living within ½ mi of an unconventional gas well were found to have an increased risk of neurological and respiratory health effects than residents living greater than ½ mi away. The risk of cancer was increased in these residents as well, with benzene and ethylbenzene as the primary hydrocarbon contributors (McKenzie et al. 2012).

## Discussion

In the 155 years since the first modern oil well was drilled in Pennsylvania, technology has evolved from the spring pole to modern rotary rigs that can drill miles into the earth (American Oil & Gas Historical Society 2015). The more recent technological advancement of horizontal hydraulic fracturing has changed the landscape of gas and oil production.

Natural gas has the potential for a smaller carbon footprint than historical fossil fuel sources; for instance, there are substantially lower emissions of nitrous oxides and carbon dioxide per Btu of energy produced compared to coal (U.S. EIA 2015). As a result of the ability to access unconventional formations, the United States is less dependent on foreign natural gas; the United States has now surpassed Russia as the world’s largest natural gas producer (Ratner and Tiemann 2015). While the influx of wells and related natural gas infrastructure has advanced the economics of some individuals and communities (API 2014), questions remain about public health and safety when a heavy industrial process is placed close to the public. The consequence of these concerns is that public support for hydraulic fracturing is declining, and the industry realizes the need to minimize risks to communities and the environment (Dittrick 2015). Setbacks are an attempt to address this need. Our paper attempts to address whether the current setback laws in three heavily drilled states within the Barnett, Marcellus, and Niobrara shale plays are sufficient to protect public health and safety.

The majority of setback distances in the areas we studied are not derived from peer-reviewed data, data driven analysis, or historical events (Fry 2013)—they are a compromise between governments, the regulated community, environmental and citizen interest groups, and landowners (COGCC 2013). In part to address the issue of setbacks, the University of Maryland School of Public Health performed an in-depth analysis of the current data, and prepared a report for the Maryland Department of the Environment and the Department of Health & Mental Hygiene. The authors recommended a minimum setback distance of 2,000 ft from well pads (Maryland Institute for Applied Environmental Health 2014). Also in 2014, the New York State Department of Health (NY DOH) published the results of a Public Health Review process. In preparing the report, the NY DOH reviewed and evaluated scientific literature, obtained input from outside public health expert consultants, engaged in field visits and discussions with health and environmental authorities in states with hydraulic fracturing activity, and communicated with multiple local, state, federal, international, academic, environmental, and public health stakeholders. The DOH report concluded that hydraulic fracturing activity has resulted in environmental impacts that are potentially adverse to public health (NY DOH 2014). As a result of this study, the Concerned Health Professionals of New York recommended a moratorium on hydraulic fracturing in New York State until it could be determined whether the potential risks could be managed (Concerned Health Professionals of New York 2014); the state subsequently banned the practice altogether (Klopott 2015). Citing similar concerns of environmental contamination, some countries, including France, Bulgaria, and Scotland have current bans and moratoria on hydraulic fracturing (Patel 2015).

In the geographic areas we studied, the most common setback distances from buildings were 300 and 500 ft with a range of 150–1,500 ft. Based on historical catastrophic events, thermal modeling, vapor cloud modeling, and air pollution data, these distances do not appear sufficient to protect public health and safety. We address each of these subsections below.

### Blowouts and Evacuations/Thermal Modeling

Blowouts can cause drill pipe, mud, cement, fracking fluids, and produced water (water that has been used in the hydraulic fracturing process) to be ejected from the bore and expelled at high pressure. These drilling materials can be followed by production waters, gases and/or petroleum. Gas well blowouts can be very dangerous since a spark can set off an explosion (Nguyen 2010). Fires can involve other equipment on the well pad, releasing additional fumes, smoke, and volatiles (Arrington 2014). If members of the public are located in the vicinity, evacuations may be required, with a safety perimeter established around the well (Wild Well Control, Inc., and Travelers Indemnity Company 2012). Historical data indicate that blowout frequency is approximately 1 per 10,000 wells (OGP 2010). Published data from the Marcellus Shale indicates a blowout risk of 0.17%, with a well barrier or integrity failure rate of 6.3% for the years 2005–2013 (Davies et al. 2014); this is consistent with the historical numbers. Well blowout preventers are intended to control the internal well pressure; however, these blowout preventers are not failsafe (Nguyen 2010).

**Figure 2:**
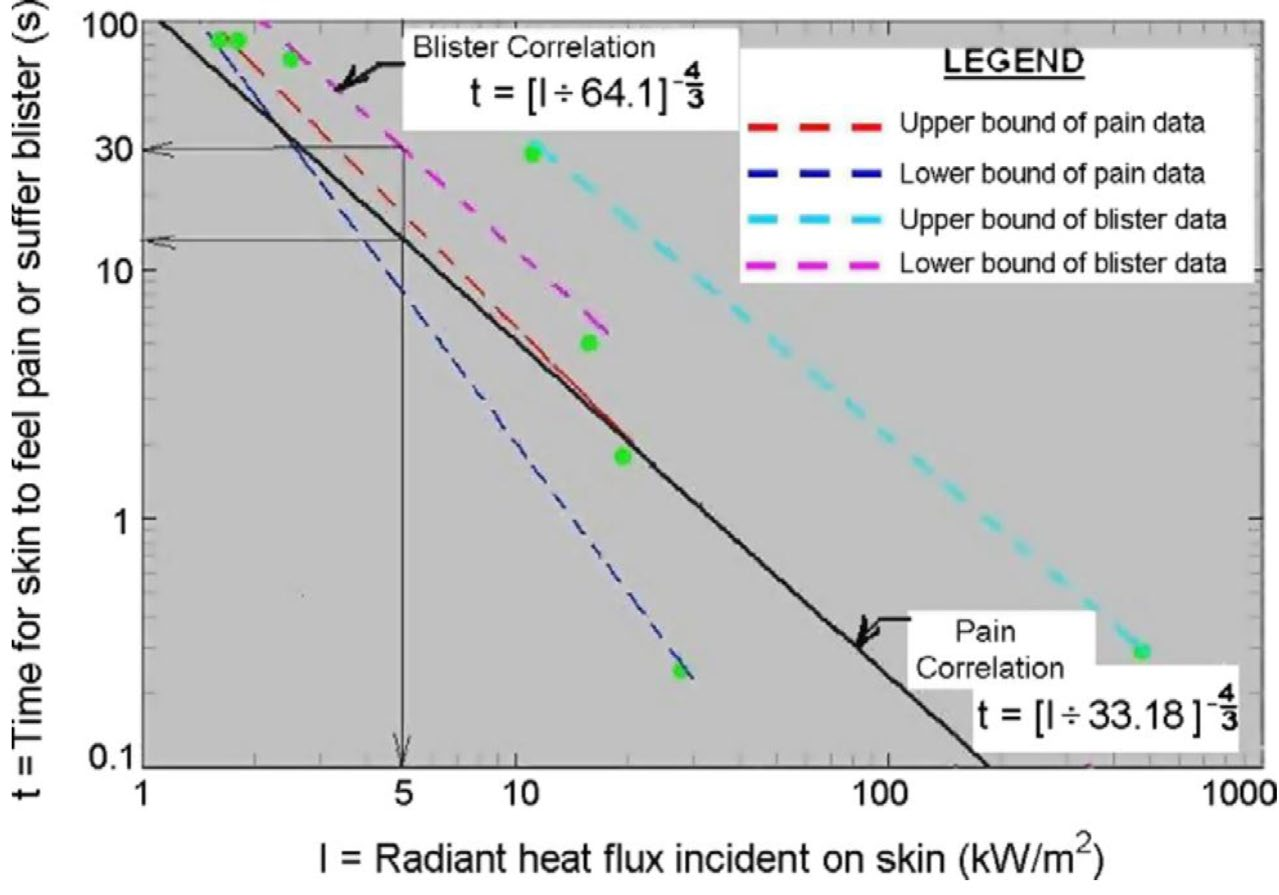
Range of experimental data on skin pain and skin burns and correlations of time for injury vs. incident radiant flux. (From Raj PK. A review of the criteria for people exposure to radiant heat flux from fires. *J Hazard Mater* 2008;159:61–71, with permission from Elsevier.)

The Federal Emergency Management Agency (FEMA) Emergency Management Institute provides recommendations for emergency planning and response (Appendix 1) (FEMA 2015). During a level 3 event within the suburban setting, special consideration must be given to gas plume concentration/dispersion, smoke, hydrogen sulfide gas, explosions, heat radiation, and effects on buildings, homes, power lines, and nearby well and gas pipelines. Once the decision to evacuate is made, it should be done quickly and efficiently, with ongoing communication and assistance to evacuees (Wild Well Control, Inc., and Travelers Indemnity Company 2012). Based on thermal modeling, at 500 ft, the thermal exposure to those evacuating would be below the NFPA standard of 5 kW/m^2^ (NFPA 2015). 2.7 kW/m^2^ at 500 ft is what firefighters encounter and up to second degree burns will occur after 30 min or less of unprotected exposure, as indicated by sunburn type at 1.4 kW/m^2^ at 30 min (Arrington 2014). API proposes a level of 6.3 kW/m^2^ for situations in which emergency actions lasting up to 30 sec may be required by people without shielding but wearing clothing (API 2007). At the common Texas setback distance of 300 ft and the Colorado outdoor recreational distance of 350 ft, based on the calculation of radiant heat flux, second degree burn blisters would be expected to form after approximately 16 sec and 22 sec, respectively (Figure 2).

In the evacuation data we collected, the average evacuation zone was 0.8 mi (range of 660–13,200 ft) and the average number of homes/families displaced was 149 (range of 3–500 per event). Two incidents required aircraft diversion, one in the Barnett Shale (Heinkel-Wolfe 2013) and one in the Marcellus Shale (Nguyen 2010). An explosion in the Barnett Shale produced a 750 ft burn crater (Heinkel-Wolfe 2013; Nguyen 2010). The sizes of the evacuation zones, the number of families displaced, and the presence of a measurable burn crater, along with the thermal modeling data above, raise several questions: Does current unconventional gas well preplanning take into account *a*) the number of people to be evacuated from an area, *b*) the time it would take to evacuate, and *c*) the route needed for evacuation? Unfortunately, this does not appear to uniformly be the case. Wolverton (2010) published an Applied Research Project for the city of Shreveport, Louisiana, focusing on the hazards, challenges, and concerns regarding emergency response and public safety in relation to natural gas wells. For this study, a literature review was performed through the National Fire Academy’s Learning Resource Center (U.S. Fire Administration 2016), search engines from the web, and published articles. Wolverton concluded that there was minimal research done on the topic of emergency response preplanning. In the Barnett shale area, some individual municipalities and gas companies develop and mail brochures to residents near gas wells, but this is not a uniform practice. Among the major challenges to responding to gas well hazards, Wolverton identified a lack of preplanning, inadequate resources, proximity to high-occupancy facilities, size of fires, and lack of training and equipment (Wolverton 2010).

During a level 3 event involving a gas well, officials should have a clear plan of notification, transportation, and evacuation routes for high-occupancy buildings. The COGCC appears to be considering this concept with the increased setback requirement for high-occupancy buildings, including schools (COGCC 2013). School evacuation protocols vary among states and districts; in general, in ideal circumstances, a fire drill evacuation is accomplished in several minutes. In an actual emergency, however, the evacuation time may be longer. For instance, after a school shooting in Connecticut, once the shelter in place was lifted, it took over 30 min to evacuate Sandy Hook Elementary School (Connecticut State Police 2013). Historical evacuation data, as well as the potential for thermal injury during an evacuation, should be taken into consideration when planning the location of a well.

### Air Pollution/Vapor Dispersion

With variable frequency, benzene levels are elevated at multiple locations in close proximity to some gas development sites (Epstein 2016). This is not unexpected, considering that benzene occurs naturally in crude petroleum in levels up to 4 g/L (WHO 2010). At issue is that the frequency of elevated levels is sufficient to present a public health risk. Benzene is released from a number of natural gas extraction processes, and has the potential for adverse human health outcomes through inhalation exposure (Finkel et al. 2013).

In 2014, Bunch et al. (2014) published results of air monitoring from 4.6 million data points (representing data from seven monitors at six locations). Using a qualitative risk-based approach, the authors concluded that shale gas production activities have not resulted in exposures to VOCs, including benzene, at levels that would pose a health concern (Bunch et al. 2014). As discussed previously in this paper, however, other air monitoring studies have found benzene exceeding recommended health-based risk levels (McCawley 2013; McKenzie et al. 2012). Also notable is that multiple studies have found an association between proximity to natural gas well sites and adverse health outcomes, including congenital defects (McKenzie et al. 2014), decreased birth weight (Stacy et al. 2015), and increased hospitalization rates (Jemielita et al. 2015). These findings lend weight to the possibility that pollution from shale gas activities could potentially precipitate adverse health effects.

Hydrogen sulfide modeling has shown toxic gas cloud dispersion beyond even the most generous setback in our states of interest (U.S. DOT 2005). Dispersion modeling has also shown carbon dioxide and carbonyl sulfide plumes extending in excess of 1 mi from drill sites (Fort Worth League of Neighborhoods 2011). H_2_S has an odor threshold of 0.01–1.5 ppm, whereupon people will begin to notice the unpleasant characteristic “rotten egg” smell. The odor becomes offensive at 3–5 ppm (U.S. Department of Labor 2015). Levels at which odor can be detected have been associated with mucosal irritation, respiratory symptoms, and need for anti-asthma drugs (ATSDR 2014). In a controlled setting, adults exposed to a range of H_2_S from 0.05 to 5 ppm experienced anxiety and compromised verbal learning performance (Fiedler et al. 2008). At the basic science level, laboratory studies have shown genotoxicity and DNA damage from H_2_S. Odor exposure is also associated with negative mood, stress, and annoyance for those living near H_2_S-producing facilities (ATSDR 2014). Combined with the VOCs, this produces a potentially new set of exposures, possibly at distances of 2 km, which have not yet been well characterized nor well studied for their accompanying health effects. For example, there are recurring reports of nose bleeds and a metallic taste in populations living near drilling activity (McCawley 2015). A survey-based ambient health effects study showed that prevalence of dermal and respiratory complaints increased with proximity to drilling activities (Rabinowitz et al. 2015) (Table 3).

**Table 3:** Prevalences of reported respiratory disease in areas near drill sites (Rabinowitz et al. 2015).

Respiratory symptoms	< 1 km (*N* = 150)	1–2 km (*N* = 150)	> 2 km (*N* = 192)
Upper respiratory [*n* (%)]	58 (39)	46 (31)	35 (18)
Lower respiratory [*n* (%)]	29 (19)	29 (19)	27 (14)

Air pollution from inadequate setbacks is of particular concern for vulnerable populations. The economically disadvantaged, people > 65 years old, and younger people with disabilities are most likely to have chronic health conditions which require institutional care (American Hospital Association 2011). In Pennsylvania, those living below the poverty line are significantly more likely to be exposed to pollution from unconventional gas wells (Ogneva-Himmelberger and Huang 2015). Children are a group that deserves special consideration, as physical vulnerabilities increase children and youth’s susceptibility to illnesses, including asthma and other respiratory ailments (USDA 2012). Children are also more vulnerable to pollutants by nature of their developmental status (Pediatric Environmental Health Specialty Units 2011). These facts bring into particular question the wisdom of granting permits for unconventional gas wells in close proximity to schools and health care facilities, where a significant number of vulnerable individuals would be expected to be located.

With regard to air pollution associated with hydraulic fracturing, current setbacks do not appear to be fully protective. Although appropriately set distances may provide some measure of safety, setbacks do not necessarily reduce risk associated with potentially hazardous air emissions. Not all emissions emanate from the point of drilling and many may originate from distances as far away from the well pad as the setback distance itself, or even beyond. For example, when measured at the same setback distance for all the processes in an active drilling operation in the West Virginia study, the benzene concentration fluctuated substantially due to the proximity of the source to the setback distance (McCawley 2013). At the highest concentration, the source (a flare) was immediately adjacent to the samplers, even though the samplers were 625 ft from the center of the well pad. In this scenario, a setback does nothing to control the location or strength of the multiple possible sources at a well site and so it cannot be considered a control at all.

Given the advantages of domestic natural gas development, the question arises as to whether the risks of hydraulic fracturing are acceptable, particularly in close proximity to the public. There are many accepted definitions and permutations of acceptable risk, depending on one’s point of view. From a business standpoint, acceptable risk is generally considered to be injury or loss from an industrial process that is considered tolerable by a society in view of the political, social, and economic cost-benefit analysis. From a scientific standpoint, the Precautionary Principle, which is endorsed by multiple national and international agencies, states that in cases of serious or irreversible threats to the health of humans or ecosystems, acknowledged scientific uncertainty should not be used as a reason to postpone preventive measures (WHO 2004). The U.S. EPA calculates both non-cancer and cancer risks from chemical exposure. Non-cancer risk is calculated by comparing the estimated daily intake of the chemical over a specific time period with the reference dose for that chemical derived for a similar period of exposure. Cancer risk is the probability that an exposed individual will develop cancer due to that exposure by age 70. For each chemical of concern, this value is calculated from the daily intake of the chemical from the site averaged over a lifetime, including a slope factor. In general, the U.S. EPA considers excess cancer risks that are below about 1 chance in 1,000,000 to be so small as to be negligible, and risks above 1 in 10,000 to be sufficiently large that some sort of remediation is preferred. The level of total cancer risk that is of concern, however, is a matter of personal, community, and regulatory judgment (U.S. EPA 2015c). Our findings represent an important case study for the science of risk assessment and public policy decisions of risk management. In the United States, risk management strategies for gas development vary widely by state, including acceptance of large-scale development (Texas, Pennsylvania, Colorado); more cautious consideration with extended controls and protections (Maryland); and outright bans (New York). The question remains as to whether society will continue to accept the level of risk associated with shale gas development given its potential benefits.

There are at least some additional actions to help to mitigate risk. The report by Wolverton (2010) highlighted the need for comprehensive planning prior to drilling. For detection of air pollution, air monitors could be placed at sensitive locations, and the sites connected to a central monitoring station by cellular phone or Wi-Fi to record air emission levels 24 hr a day. When the desired levels are exceeded, engineers would investigate to seek the source and report not only the cause, but also the steps taken to prevent a recurrence. Monitoring of all pertinent hazards could be considered for future regulations in conjunction with setbacks (Ziemkiewicz et al. 2014). In addition, the standard method of measuring air quality, using periodic 24-hr averages, does not accurately reflect the intensity, frequency or duration of meaningful exposure to the pollutants associated with the hydraulic fracturing process (Brown et al. 2014). Another factor to consider is well density. Risk calculations for environmental hazards are often based on measurements from a single source (U.S. EPA 2015a). In today’s hydraulic fracturing environment, however, public exposure can come from multiple sources–either from multiwell pads or single well pads in proximity to one another. Simultaneous operations can introduce multiple hazards carrying additional risks (Boquist 2014). Applying accurate and comprehensive measurement techniques, along with mitigation factors, could allow selection of a setback based on the level of control exercised and maintained rather than on arbitrary distances set by legislative compromise.

## Limitations

Our present study has some limitations. There are over 20 shale plays in the lower 48 United States (U.S. EIA 2011a); by confining our study to 3 shale plays, the scope of data was narrowed. We also limited our study to well sites. Excluding pipelines limited data on explosions and evacuations (Riordan Seville 2014), and excluding compressor stations restricted air pollution results (Shogren 2011). An inclusive study of the outcomes outlined in this study would include the wells and the potential contribution from necessary accompanying infrastructure.

Some of the evacuation data and noise complaint cases were gathered from media reports, which can introduce reporting errors and/or bias. Whenever possible, we evaluated information from multiple sources to determine consistency. Not all well blowouts required evacuations or had evacuation data available; for our analysis, we focused on those blowouts for which we could report an evacuation distance and/or number of families displaced.

Our air pollution analysis is by no means comprehensive. In the past several years, more data have emerged regarding air pollution related to hydraulic fracturing. Studies have varied in methods of collection and analysis; however, multiple studies show air pollutants at levels which raise health concerns (Shonkoff et al. 2014). We focused on those studies which raised concern regarding benzene and H_2_S levels; a more thorough air pollution analysis would include nitrogen oxides, ozone, particulate matter, and the spectrum of VOCs (Shonkoff et al. 2014). In addition, benzene levels are characterized by high variability, which can result in inconsistencies within and between studies. Compounding the difficulty is the fact that air pollution varies widely, and there is an unmet need to study the episodic nature of air pollutant emissions.

Our thermal modeling was based on an average gas well. At each site, it is crucial to take into account the local geography, weather patterns, engineering specifics of each particular well, and nearby structures, which was not feasible for the purposes of this study.

## Conclusion

Current natural gas well setbacks in the Barnett Shale of Texas, the Marcellus Shale of Pennsylvania, and the Niobrara Shale of Colorado cannot be considered sufficient in all cases to protect public health and safety. Based on historical evacuations and thermal modeling, people within these setback distances are potentially vulnerable to thermal injury during a well blowout. According to air measurements and vapor dispersion modeling, the same populations are susceptible to benzene and hydrogen sulfide exposure above health-based risk levels. Texas, Pennsylvania, and Colorado should consider adopting more generous setback distances, particularly in reference to vulnerable populations; however, distance is not an absolute measure of protection. Unfortunately, there is no defined setback distance that assures safety. As mitigation technology advances, current setback distances may eventually be sufficient to protect the public. Unfortunately, current mitigations are not fail-safe, and each has its limitations (U.S. Forest Service 2011). The results of our analysis based on three states suggest that assuming the threat posed to health originates from either the center of the drill pad or some small distance surrounding it requires reevaluation. A combination of a reasonable setback with accompanying controls on all aspects of the process is the best method for reducing the potential threats to public health.

**Appendix 1:** FEMA EMI recommendations for emergency planning and response (FEMA 2015).

Emergency planners should anticipate both active and passive resistance to the planning process and develop strategies to manage these obstacles.	Preimpact planning should address all hazards to which the community is exposed.	Preimpact planning should elicit participation, commitment, and clearly defined agreement among all response organizations.	Preimpact planning should be based upon accurate assumptions about the threat, typical human behavior in disasters, and likely support from external sources such as state and federal agencies.	EOPs should identify the types of emergency response actions that are most likely to be appropriate, but encourage improvisation based on continuing emergency assessment.	Emergency planning should address the linkage of emergency response to disaster recovery and hazard mitigation.	Preimpact planning should provide for training and evaluating the emergency response organization at all levels—individual, team, department, and community.	Emergency planning should be recognized as a continuing process.
